# Correction: Apa et al. Ultrasonic Surgical Aspirator in Intramedullary Spinal Cord Tumours Treatment: A Simulation Study of Vibration and Temperature Field. *Bioengineering* 2025, *12*, 842

**DOI:** 10.3390/bioengineering13070805

**Published:** 2026-07-14

**Authors:** Ludovica Apa, Sergio Di Nitto, Mauro Palmieri, Pietro Familiari, Emanuele Rizzuto, Zaccaria Del Prete

**Affiliations:** 1 Department of Mechanical and Aerospace Engineering, University of Rome La Sapienza, Via Eudossiana 18, 00184 Rome, Italy; sergio.dinitto@outlook.com (S.D.N.); emanuele.rizzuto@uniroma1.it (E.R.); zaccaria.delprete@uniroma1.it (Z.D.P.); 2 Department of Human Neurosciences, Division of Neurosurgery, Policlinico Umberto I University Hospital, Sapienza University of Rome, 00157 Rome, Italy; mauro.palmieri@uniroma1.it (M.P.); pietro.familiari@uniroma1.it (P.F.)

Sergio Di Nitto was not included as an author in the original publication [1]. The corrected Author Contributions statement appears here. The authors state that the scientific conclusions are unaffected. This correction was approved by the Academic Editor. The original publication has also been updated.

**Author Contributions:** Conceptualization, L.A., M.P., P.F., E.R. and Z.D.P.; methodology, L.A. and M.P.; software, L.A. and S.D.N.; validation, L.A., M.P., P.F., E.R. and Z.D.P.; formal analysis, L.A.; investigation, L.A.; resources, L.A., E.R. and Z.D.P.; data curation, L.A., S.D.N. and M.P.; writing—original draft preparation, L.A.; writing—review and editing, L.A., S.D.N., M.P., P.F., E.R. and Z.D.P.; visualization, L.A.; supervision, P.F. and Z.D.P.; project administration, Z.D.P.; funding acquisition, Z.D.P. All authors have read and agreed to the published version of the manuscript.
